# Phase I trial of HuMax-IL8 (BMS-986253), an anti-IL-8 monoclonal antibody, in patients with metastatic or unresectable solid tumors

**DOI:** 10.1186/s40425-019-0706-x

**Published:** 2019-09-05

**Authors:** Marijo Bilusic, Christopher R. Heery, Julie M. Collins, Renee N. Donahue, Claudia Palena, Ravi A. Madan, Fatima Karzai, Jennifer L. Marté, Julius Strauss, Margaret E. Gatti-Mays, Jeffrey Schlom, James L. Gulley

**Affiliations:** 1 Genitourinary Malignancies Branch, Center for Cancer Research, National Cancer Institute, National Institutes of Health, Bethesda, MD USA; 2 Laboratory of Tumor Immunology and Biology, Center for Cancer Research, National Cancer Institute, National Institutes of Health, Bethesda, MD USA; 3 Medical Oncology Service, Center for Cancer Research, National Cancer Institute, National Institutes of Health, Bethesda, MD USA; 4 Precision Biosciences, Durham, NC USA

**Keywords:** Interleukin-8 (IL-8), Metastatic cancer, Solid tumor, HuMax-IL8, BMS-986253, Monoclonal antibody, Immunotherapy, Clinical trial, Immune assays

## Abstract

**Background:**

HuMax-IL8 (now known as BMS-986253) is a novel, fully human monoclonal antibody that inhibits interleukin-8 (IL-8), a chemokine that promotes tumor progression, immune escape, epithelial-mesenchymal transition, and recruitment of myeloid-derived suppressor cells. Studies have demonstrated that high serum IL-8 levels correlate with poor prognosis in many malignant tumors. Preclinical studies have shown that IL-8 blockade may reduce mesenchymal features in tumor cells, making them less resistant to treatment.

**Methods:**

Fifteen patients with metastatic or unresectable locally advanced solid tumors were enrolled in this 3 + 3 dose-escalation trial at four dose levels (4, 8, 16, or 32 mg/kg). HuMax-IL8 was given IV every 2 weeks, and patients were followed for safety and immune monitoring at defined intervals up to 52 weeks.

**Results:**

All enrolled patients (five chordoma, four colorectal, two prostate, and one each of ovarian, papillary thyroid, chondrosarcoma, and esophageal) received at least one dose of HuMax-IL8. Eight patients had received three or more prior lines of therapy and five patients had received prior immunotherapy. Treatment-related adverse events occurred in five patients (33%), mostly grade 1. Two patients receiving the 32 mg/kg dose had grade 2 fatigue, hypophosphatemia, and hypersomnia. No dose-limiting toxicities were observed, and maximum tolerated dose was not reached. Although no objective tumor responses were observed, 11 patients (73%) had stable disease with median treatment duration of 24 weeks (range, 4–54 weeks). Serum IL-8 was significantly reduced on day 3 of HuMax-IL8 treatment compared to baseline (*p* = 0.0004), with reductions in IL-8 seen at all dose levels.

**Conclusions:**

HuMax-IL8 is safe and well-tolerated. Ongoing studies are evaluating the combination of IL-8 blockade and other immunotherapies.

**Trial registration:**

NCTN, NCT02536469. Registered 23 August 2015, https://clinicaltrials.gov/ct2/show/NCT02536469?term=NCT02536469&rank=1.

**Electronic supplementary material:**

The online version of this article (10.1186/s40425-019-0706-x) contains supplementary material, which is available to authorized users.

## Background

Interleukin-8 (IL-8) is a pro-inflammatory chemokine from the CXC family; it is also known as CXCL8. It mediates biologic effects by binding to two cell-surface G protein-coupled receptors, IL-8RA (CXCR1) and IL-8RB (CXCR2), which are expressed on neutrophils, monocytes, endothelial cells, and cancer cells [1–4]. Expression of IL-8 is regulated by chemical and environmental stresses such as chemotherapies and hypoxia and by inflammatory signals [5, 6]. IL-8 mediates the activation and chemotaxis of immune cells leading to chronic inflammation [7, 8]. IL-8 is also frequently overexpressed in many human carcinomas, including breast, colon, cervical, gastric, lung, and ovarian [9–13]. Studies have shown a direct correlation between serum IL-8 levels and disease progression [14, 15]. IL-8 has also been linked to tumor stem cell-like properties or “stemness,” including self-renewal, differentiation, and proliferative potential. Breast cancer cells with elevated aldehyde dehydrogenase activity, a marker of breast cancer stem cells, express high levels of IL-8 receptor (IL-8R) [16]. IL-8 functions in both a paracrine and autocrine mode within the tumor microenvironment (TME) to foster tumor progression, invasiveness, and metastasis [4]. IL-8 signaling is known to influence the TME and promote cancer progression by (a) inducing the angiogenic response of endothelial cells, (b) recruiting neutrophils and myeloid-derived suppressor cells (MDSCs) to the tumor bed, (c) facilitating the proliferation, survival, and migration of tumor cells, and (d) promoting epithelial-mesenchymal transition (EMT) [17–22].

Induction of the IL-8/IL-8R axis has also been shown to increase levels of brachyury, a transcription factor overexpressed in a variety of carcinomas but absent in the majority of normal adult tissues [23]. Brachyury has been shown to regulate EMT in human carcinoma cells and to induce mechanisms of tumor resistance to chemotherapy and radiation [24–26]. Conversely, antibody blockade of IL-8/IL-8R markedly reduces the expression of mesenchymal markers, decreases recruitment of MDSCs, and enhances immune-mediated lysis of tumor cells [23, 27]. These data suggest that pharmacologic inhibition of IL-8 is a rational approach for the treatment of a variety of malignancies.

HuMax-IL8 (previously known as BMS-986253) is a fully human IgG1 kappa monoclonal antibody that binds to free IL-8 [4]. The safety and efficacy of HuMax-IL8 monotherapy was tested in a phase I/II clinical trial in patients with palmoplantar pustulosis, a rare chronic inflammatory skin disorder. The drug was well tolerated and effective in reducing disease activity at doses of 0.15 to 8 mg/kg IV. The maximum tolerated dose (MTD) was not reached. The antibody had a half-life of approximately 11 days [7].

This phase I study evaluated the safety and tolerability of HuMax-IL8, as well as changes in serum IL-8 levels, peripheral immune subsets, and circulating tumor cells (CTCs) in patients with incurable metastatic or unresectable solid tumors. This is the first study to evaluate this agent in patients with cancer and this study utilized higher doses of the agent than had been previously tested in any disease.

## Patients and methods

### Eligibility

Eligible patients had incurable metastatic or unresectable, locally advanced malignant solid tumors that were evaluable or measurable. They must have completed or had disease progression on at least one prior line of disease-appropriate therapy for metastatic disease, or not be a candidate for therapy with proven efficacy for their disease. There had to be a minimum of 4 weeks from any prior chemotherapy, immunotherapy (6 weeks if immune checkpoint inhibitor), and/or radiation**,** but patients with colorectal cancer were allowed to continue on maintenance capecitabine and/or bevacizumab. Patients were required to be ≥18 years of age, have an Eastern Cooperative Oncology Group (ECOG) performance status of ≤1, no other malignancies within 12 months, no significant medical illnesses or autoimmune diseases, and acceptable hematologic parameters and organ function. No local or systemic steroids except for physiologic replacement doses were allowed within 2 weeks of enrollment. Patients were excluded if they had untreated central nervous system metastases or local treatment of brain metastases within the previous 6 months, HIV, or chronic hepatitis B or C infection.

### Assessment of toxicities

Toxicity was evaluated according to National Cancer Institute Common Terminology Criteria for Adverse Events v4.0. A dose-limiting toxicity (DLT) was defined as any grade ≥ 4 hematologic toxicity or any grade ≥ 3 nonhematologic toxicity, with minor exceptions, or any grade ≥ 3 allergic reaction or autoimmune reaction that was definitely, probably, or possibly related to the administration of HuMax-IL8.

### Study design

This was a single-institution, open-label, phase I clinical study intended to determine the safety and MTD of HuMax-IL8 at four dose levels (4, 8, 16, and 32 mg/kg) IV every 2 weeks with each cycle being 28 days. Accrual of up to 24 patients in the dose-escalation phase and an additional 20 patients in the dose-expansion phase were initially planned.

**Fig. 1:**
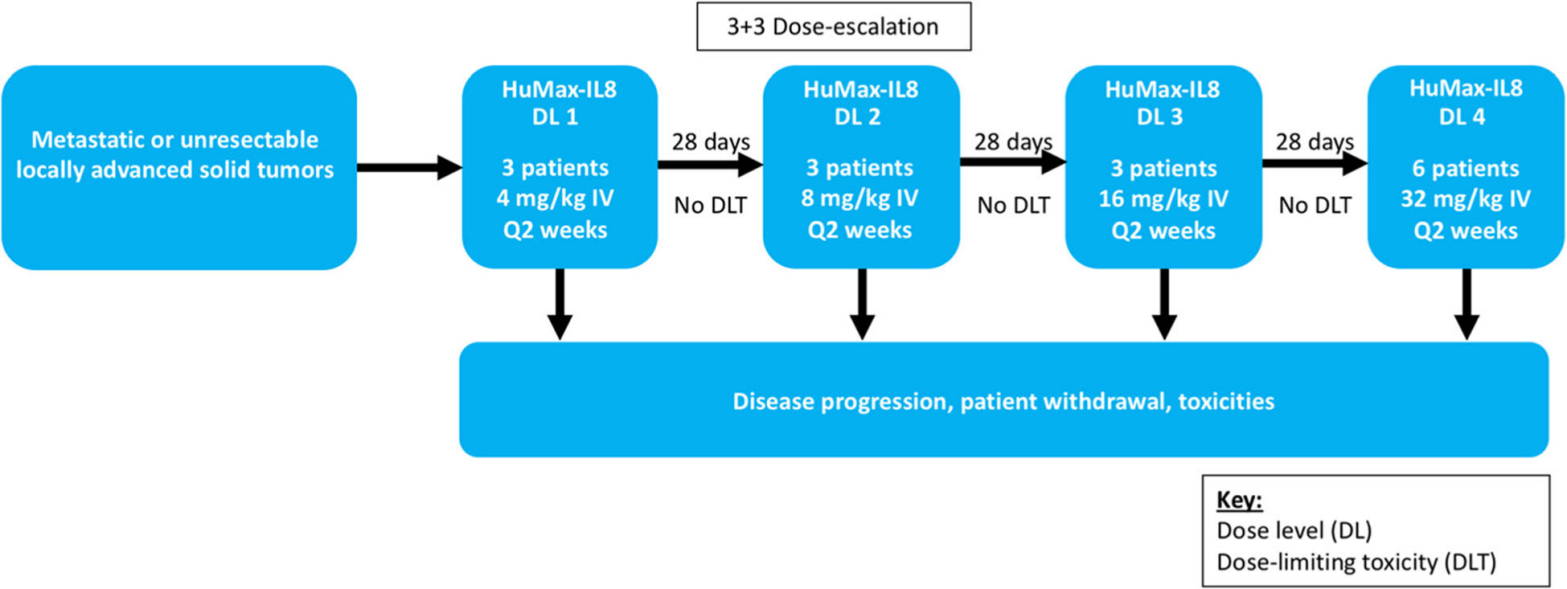
Trial schema. Trial schema with dose-escalation strategy

Enrollment in each dose cohort proceeded in the standard 3 + 3 scheme with sequential cohorts of patients (three to six patients per cohort) (Fig. 1). The decision to escalate to the next dose level was based on the observation of DLTs during the 28-day period following the first dose of HuMax-IL8. The MTD was defined as one dose level below the maximum administered dose.

Tumor responses were evaluated by CT of the chest/abdomen/pelvis (with optional MRI or PET scan) at baseline and then every 2 months. Treatment was continued until disease progression, unacceptable adverse events (AEs), or withdrawal from the study. The safety and efficacy of the study drug were assessed until the end of treatment or for a maximum of 52 weeks.

Response to treatment was measured by combined immune-related response criteria (irRC) and RECIST v1.1. The main modifications from RECIST 1.1 were (a) a requirement for confirmation of both progression and response by imaging at least 4 weeks after initial imaging and (b) not automatically calling the appearance of new lesions progressive disease if the total measurable tumor burden had not met criteria for progressive disease. In the spirit of the irRC, any new lesions in these subjects required confirmation of the new lesion on repeat imaging at least 4 weeks later to ensure that new lesions were not immune-related phenomena [28].

### Immune assays

Blood samples for immune-cell assessment were collected at baseline and on days 3 and 15 of cycle 1, prior to cycles 2 and 3, and then every other cycle. Blood samples for analysis of CTCs were collected at baseline, prior to cycles 2 and 3, and then every other cycle (for details see Additional file 1).

### Statistical analysis

Summary statistics were used to describe demographic data and baseline performance status characteristics. Results of safety evaluations were tabulated and displayed by dose level. Only exploratory statistical analyses were performed due to the limited number of subjects receiving each dose level. Descriptive statistics were examined for indications of dose-related toxicity. Immunological parameters were mainly analyzed descriptively and displayed in graphic format using GraphPad Prism (GraphPad Software, La Jolla, CA). For changes in peripheral immune subset and cytokine analyses, *p* values were calculated using the Wilcoxon matched pairs signed rank test. Subsets with a potentially biologically relevant change were defined as those with a *p* < 0.05, majority of patients > 25% change, differences in medians of pre- vs. post-therapy > 0.01% of peripheral blood mononuclear cells (PBMCs), and a frequency > 0.01% of PBMCs. The Mann-Whitney test was used to evaluate differences in immune subsets at baseline. Fisher’s exact test was used to evaluate the proportion of patients with IL-8 levels under a given threshold.

## Results

### Patient population

Fifteen patients were enrolled between August 2015 and May 2016 at the National Cancer Institute in Bethesda, Maryland. Five patients had chordoma, four had colorectal cancer, two had prostate cancer, and one each had ovarian, papillary thyroid, chondrosarcoma, and esophageal cancer. The patients were predominantly white (80%) with a mean age of 59.7 years (range 39–73). Eight patients had received three or more prior lines of therapy. Prior immunotherapy regimens ranged from 0 to 3 (median 0), which included therapeutic cancer vaccines (5 patients), cytokines (2 patients), and checkpoint inhibitors (2 patients). The median treatment duration was 24 weeks (range, 4–54 weeks). Baseline characteristics are summarized in Additional file 1: Table S1.

### Safety

Safety and efficacy were assessed until the end of treatment or for a maximum of 52 weeks. No DLTs were observed and no severe adverse events (SAEs) related to treatment with HuMax-IL8 occurred. MTD was not identified through 32 mg/kg, supporting 32 mg/kg of HuMax-IL8 IV every 2 weeks as the recommended phase II dose. Grade 1 or 2 treatment-related AEs occurred in five patients (Table 1).

**Table 1:** Treatment-related adverse events

	Dose (mg/kg)	Grade 1, N (%)	Grade 2, N (%)
Nausea	4 and 8	2 (13.3)	0
Headache	4	1 (6.7)	0
Chills	8	1 (6.7)	0
Throat tightness	8	1 (6.7)	0
Decreased WBC	16	1 (6.7)	0
Fatigue	32	1 (6.7)	2 (13.3)
Constipation	32	1 (6.7)	0
Hypersomnia	32	0	2 (13.3)
Hypophosphatemia	32	0	2 (13.3)

All adverse events at least possibly attributed to study drug are shown. There were no grade 3 or grade 4 treatment-related adverse events. Adverse event grade is according to National Cancer Institute Common Terminology Criteria for Adverse Events v4.0

*kg* kilogram

*mg* milligram

*WBC* white blood cell

Two SAEs were reported in one patient receiving 4 mg/kg of HuMax-IL8, and five grade 3 SAEs were reported in three patients receiving 8 mg/kg, 16 mg/kg, and 32/mg, respectively. The grade 3 SAEs were pain, increased blood alkaline phosphatase, abdominal infection, hyponatremia, and a fall. The most common AEs were constipation (33.3%), nausea (26.7%) and anemia (26.7%). AEs leading to discontinuation of HuMax-IL8 were reported in three patients (20%); they included grade 3 increased blood alkaline phosphatase in one patient, grade 2 increased blood creatinine and grade 3 hypertension in one patient, and grade 3 fall and back pain in one patient, none of which were considered to be related to the study drug.

### Pharmacokinetics

Noncompartmental analysis characterized the pharmacokinetic parameters for HuMax-IL8. Maximum concentrations were observed within 1–5 h across the doses evaluated following the 1-h infusion. Both the maximum concentration (C_max_) and area under the curve geometric mean of HuMax-IL8 demonstrated linear increases in exposure for the tested doses (Additional file 1: Table S2). Following multiple (e.g., every 2 weeks) administration of HuMax-IL8 at 4, 8, 16, and 32 mg/kg doses, steady-state pharmacokinetics had not been fully achieved by day 15 after two doses; hence there is insufficient data to report clearance and half-life using the noncompartmental method. Time dependencies in exposure have not been determined to date.

### Response to therapy

Evaluation of clinical benefit (best overall response and progression-free survival) was an exploratory objective in this study. Nine patients with stable disease came off study due to patient preference (the majority opting for another line of therapy), and six came off study due to disease progression. As shown in Table 2, best response (per RECIST v1.1) was stable disease observed in 11 patients (73.3%). The progression-free survival rate at 5.5 months was 53.3%. Time on study ranged from 2 to 13 months.

**Table 2:** Best overall response

a						
	DL 1 4 mg/kg *N* = 3	DL 2 8 mg/kg *N =* 3	DL 3 16 mg/kg *N* = 3	DL 4 32 mg/kg *N* = 6	Overall *N* = 15	
Stable disease (N, %)	3 (100%)	2 (67%)	2 (67%)	4 (67%)	11 (73%)	
Progressive disease (N, %)	0 (0%)	1 (33%)	1 (33%)	2 (33%)	4 (27%)	
b						
Patient #	Tumor type	Dose (mg/kg)	Time on study (months)*	Best response	Off treatment reason	IL-8 level decrease
1	Colorectal	4	3.5	SD	Patient choice	
2	Prostate	4	2	SD	Patient choice	x
3	Chordoma	4	8	SD	Patient choice	
4	Chordoma	8	13	SD	Patient choice	
5	Colorectal	8	1.5	PD	Progression	
6	Esophageal	8	7	SD	Progression	x
7	Chondrosarcoma	16	1	PD	Progression	x
8	Chordoma	16	5.5	SD	Progression	
9	Chordoma	16	5.5	SD	Patient choice	x
10	Papillary thyroid	32	5.5	SD	Patient choice	x
11	Colorectal	32	4.5	SD	Patient choice	x
12	Prostate	32	8	SD	Patient choice	x
13	Colorectal	32	7	SD	Patient choice	x
14	Ovarian	32	1.5	PD	Progression	x
15	Chordoma	32	2	PD	Progression	x

Best overall response. **a** Best overall response by dose level and overall are shown. **b** Responses are shown by tumor type and dose level in addition to time on study and reason for withdrawal from study treatment. Three patients had adverse events leading to their decision to come off the study. Decreases in IL-8 are also noted

*DL* dose level; *IL-8* interleukin-8; *SD* stable disease; *PD* progressive disease

### Immune assays

**Fig. 2:**
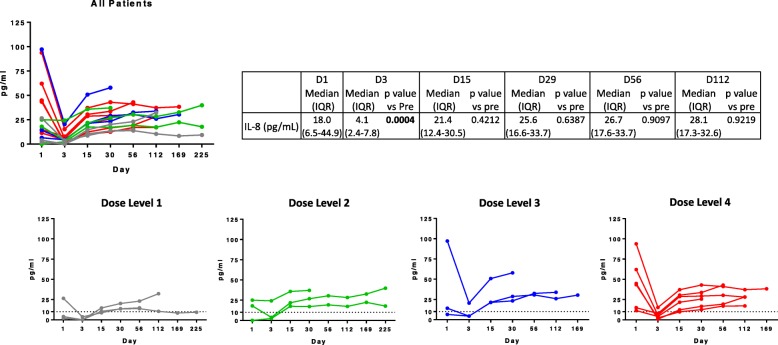
Serum IL-8 levels pre- and post-treatment with HuMax-IL8. The human IL-8 ELISA kit was used to measure free serum IL-8 levels. Reductions in serum IL-8 levels were observed at all dose levels and were significantly reduced on day 3 compared to pre-treatment (*p* = 0.0004)

Reductions in serum IL-8 levels were observed at all dose levels (Fig. 2). Serum IL-8 was significantly reduced on day 3 after HuMax-IL8 compared to baseline (*p* = 0.0004). Overall, 10/15 (67%) patients experienced a decrease in IL-8 levels, while those without a decrease in IL-8 had lower IL-8 levels at baseline (< 25 pg/mL). Prior to therapy, 11/15 (73.3%) patients had IL-8 levels > 10 pg/mL, while on day 3 after HuMax-IL8 only 3/15 (20%) patients had IL-8 levels > 10 pg/mL (*p* = 0.0092). There was a trend toward a prolonged reduction in IL-8 levels at the highest dose level (32 mg/kg), which was associated with time on study, with 4/6 patients maintaining IL-8 levels lower than baseline for at least 112 days. No significant changes were noted in the additional cytokines (IFN-γ, IL-10, IL-12p70, IL-1b, IL-2, IL-6, or TNF-α) or soluble factors (sCD27 and sCD40L) examined.

In this study, we also analyzed 123 different immune cell subsets in PBMCs prior to therapy (day 1) and on days 3, 15, 29, 56, and 112 of therapy where samples were available (Additional file 1: Table S3). There were no statistically significant changes in any immune subsets post- vs. pre-therapy at any of the time points evaluated. There were also trends in the change of specific subsets after therapy and best overall response; patients with progressive disease experienced decreases in CD4+ T cells, CD4+ T cells expressing Tim3, CD8+ T cells, CD8 T cells expressing CTLA4, central memory CD8+ T cells, B cells, B cells expressing PD-L1, and regulatory T cells (Tregs).

Thirty-nine samples from 13 patients were analyzed using Epic Science’s CTC enumeration assay; three samples were not evaluable due to DAPI < 1.5 million. Prior to therapy, CTCs (≥ 1 CTC/mL) were detected in 6/12 evaluable patients (Additional file 1: Figure S1a); nine of these patients were also evaluated for CTCs after HuMax-IL8, and CTCs were notably decreased in two of them (#3 and #7). CTCs decreased in patient #3 from 12.3 CTCs/mL prior to therapy to 1 CTC/mL on day 15, and were undetectable on days 30, 56, and 84. In patient #7, CTCs decreased from 21.8 CTCs/mL prior to therapy to 2.3 CTCs/mL on day 56; the CTCs observed after therapy demonstrated nuclear fragmentation or condensation characteristic of apoptosis (Additional file 1: Figure S1b).

## Discussion

IL-8 is overexpressed in multiple cancer types where it promotes the acquisition of mesenchymal features, stemness, resistance to therapies, and the recruitment of immune-suppressive cells to the tumor site. Through an autocrine feedback loop, IL-8 maintains the mesenchymal phenotype of tumor cells by further upregulating IL-8 and IL-8R, as well as inducing adjacent cells to undergo EMT in a paracrine mode within the tumor TME [4]. Studies in preclinical models and clinical trials have shown that antibody blockade of IL-8 produces positive effects in both non-malignant inflammatory conditions and cancer [7, 23, 27]. We have previously shown that HuMax-IL8 can revert mesenchymalization in triple negative breast cancer models both in vitro and in vivo as well as significantly decrease the recruitment of polymorphonuclear MDSCs at the tumor site, an effect substantiated when used in combination with docetaxel. HuMax-IL8 was also shown to enhance the susceptibility of breast cancer cells to immune-mediated lysis with natural killer (NK) and antigen-specific T cells in vitro, thus providing preclinical rationale for using HuMax-IL8 in combination with chemotherapy or immune-based therapies [27].

This is the first trial to investigate the effects of IL-8 blockade in patients with advanced or metastatic solid tumors. The primary endpoints were met as there were no DLTs and MTD was not reached. HuMax-IL8 is a well-tolerated drug with an acceptable safety profile for further clinical development. This trial was not powered to evaluate time to progression or overall survival. Since this trial enrolled multiple tumor types, it is difficult to compare these values to historical controls. Although no patients had an objective response, 73.3% achieved stable disease as best overall response and 53.3% remained progression-free for at least 5.5 months, indicating some patients may have derived clinical benefit. The majority of patients were heavily pretreated and it is possible that observed disease stability is characteristic of their slow-progressing disease. Nine patients with stable disease came off study in order to start another line of therapy.

Secondary and exploratory analyses evaluated changes in cytokines, immune cells, and CTCs associated with IL-8 blockade. Decreases in serum IL-8 levels were seen at all dose levels, with prolonged decreases at higher dose levels associated with a longer time on study. Several patients on this trial had surprisingly low IL-8 levels at baseline, which may make the data difficult to interpret. In addition, IL-8 levels were only measured every 2 weeks (prior to the next dose) except in cycle 1 during which IL-8 levels were measured at baseline and again on day 3, which helps explain the significant decrease in IL-8 levels noted only after the first dose. It is possible that similar serum IL-8 reductions occurred after subsequent doses but were not captured due to the timing of blood sample collection. It is also possible that although no significant changes were seen within the 123 immune cell subsets evaluated in PBMCs, this does not accurately reflect changes occurring within the tumors themselves or maybe IL**-**8 inhibition alone is insufficient to meaningfully affect immune cell subsets. No biopsies were required in this phase I study, so this information remains unknown. There were no changes in MDSCs or neutrophils in peripheral blood. CTCs did not correlate with patient outcomes. Heterogeneity of patient and tumor characteristics also makes the immune assays and CTCs difficult to interpret.

The dose-expansion phase of this trial was not conducted due to a change in the development strategy, unrelated to safety, following the decision to evaluate potential synergetic combination strategies given the safety and potential clinical benefit seen with monotherapy and preclinical rationale. Preclinical studies have also shown that inhibition of the IL-8/IL-8R pathway in a mouse sarcoma model could work synergistically with checkpoint inhibition as a means of decreasing immunosuppression within the TME [17]. In small cohorts of patients with melanoma and non-small cell lung cancer, decreases in serum IL-8 levels have been associated with response to anti-PD-1 therapy [29]. In the patients who responded to anti-PD-1 therapy there was a significant decrease in serum IL-8 levels, and at the time of progression there was a significant increase. Early changes in serum IL-8 levels (2–4 weeks after treatment initiation) were strongly associated with response and longer overall survival. There is an ongoing phase I/II clinical trial evaluating BMS-986253 plus nivolumab in patients with advanced malignancies (NCT03400332). Other therapeutic combinations also demonstrate potential. IL-8 signaling has been implicated in regulating the transcriptional activity of the androgen receptor, underpinning the transition to an androgen-independent proliferation of prostate cancer cells [30]. In addition, stress- and drug-induced IL-8 signaling has been shown to confer chemotherapeutic resistance in cancer cells. Therefore, inhibiting the effects of IL-8 signaling may be a significant therapeutic intervention in targeting the TME [3], and combinations with androgen blockade, chemotherapy, and other agents could be explored. A phase I/II clinical trial is evaluating intermittent androgen-deprivation therapy plus nivolumab with and without BMS-986253 in men with hormone-sensitive prostate cancer (NCT03689699), which may help assess the interaction of androgen and IL-8. IL-8 blockade may also have a role as an adjunct to cancer therapy to decrease rash associated with epidermal growth factor receptor (EGFR) inhibitors. A study showed that concomitant local repeat doses of a neutralizing human antibody against IL-8 reduced rash in patients receiving EGFR inhibitors, likely due to decreased neutrophil chemotaxis with the decrease in IL-8 signaling [31]. The acceptable safety profile and its potential for combination with different agents make HuMax-IL8 (BMS-986253) a promising agent for ongoing and future studies.

## Conclusions

HuMax-IL8 monotherapy is well tolerated and associated with significant decreases in serum IL-8 across all doses tested. Prolonged decreases of serum IL-8 levels were observed at higher doses of HuMax-IL-8 and were associated with a longer time on study. These data have informed combining this drug with checkpoint inhibitors and other therapies to evaluate the potential for synergetic activity in selected patient populations.

## Additional file

Additional file 1: **Table S1.** Patient demographics. **Table S2.** Pharmacokinetic analyses. **Table S3.** Immune subset analyses**. Fig. S1** Circulating tumor cells (CTC). **a** Twelve patients had evaluable whole blood samples for CTC analysis. Two patients had CTCs > 10 CTC/mL at baseline, which then decreased significantly. **b** The immunofluorescence image shows that in patient #7, the CTCs remaining at progression were apoptotic. (DOCX 483 kb)

## Data Availability

The datasets used and/or analyzed during the current study are available from the corresponding author on reasonable request.

## Abbreviations

AEs: adverse events
CI: confidence interval
CTCs: circulating tumor cells
DLT: dose-limiting toxicity
ECOG: Eastern Cooperative Oncology Group
EGFR: epidermal growth factor receptor
EMT: epithelial-mesenchymal transition
IL-8: Interleukin-8
IL-8R: IL-8 receptor
irRC: immune-related response criteria
MDSCs: myeloid-derived suppressor cells
MTD: maximum tolerated dose
NK: natural killer
PBMCs: peripheral blood mononuclear cells
PD-1: programmed cell death-1
RECIST: Response Evaluation Criteria in Solid Tumors
SAEs: severe adverse events
TME: tumor microenvironment
Tregs: regulatory T cells
