# Supplementary material for: Phase I trial of HuMax-IL8 (BMS-986253), an anti-IL-8 monoclonal antibody, in patients with metastatic or unresectable solid tumors
Source: J Immunother Cancer. 2019 Sep 5;7:240. doi: 10.1186/s40425-019-0706-x (PMC6729083; doi:10.1186/s40425-019-0706-x)
Supplement: Supplementary file 1 — Table S1. Patient demographics. Table S2. Pharmacokinetic analyses. Table S3. Immune subset analyses. Fig. S1 Circulating tumor cells (CTC). a Twelve patients had evaluable whole blood samples for CTC analysis. Two patients had CTCs > 10 CTC/mL at baseline, which then decreased significantly. b The immunofluorescence image shows that in patient #7, the CTCs remaining at progression were apoptotic. (DOCX 483 kb) [file 40425_2019_706_MOESM1_ESM.docx]

**“Phase I trial of HuMax-IL8 (BMS-986253), an anti-IL-8 monoclonal antibody, in patients with metastatic or unresectable solid tumors,” Bilusic et al.**

**Supplemental Methods**

*Serum IL-8, cytokine, and soluble factor levels*

Serum samples were collected in serum separator tubes, spun down, and stored at -80°C. Serum IL-8 levels were determined using the human IL-8 ELISA kit (RayBio, Norcross, GA) according to the manufacturer’s instructions. A competition assay confirmed that this assay does not react with IL-8 bound to HuMax-IL8 and only detects free IL-8. Serum levels of IFN-γ, IL-12p70, IL-1b, IL-2, IL-10, IL-6, and TNF-α were assayed using a multiplex cytokine/chemokine kit (Meso Scale Discovery, Gaithersburg, MD). Soluble CD27 and soluble CD40L were measured according to the manufacturer’s instructions using commercial ELISA kits (eBioscience, San Diego, CA).

*Peripheral immune-cell subsets*

Peripheral blood mononuclear cells (PBMCs were separated by Ficoll-Hypaque density gradient separation, washed three times, and cryopreserved in 90% heat-inactivated human AB serum and 10% DMSO in liquid nitrogen at a concentration of 1 x 10^7^ cells/mL until assayed. Multicolor flow cytometry was performed on cryopreserved PBMCs to identify 123 immune subsets, including nine classic subsets (CD4+ and CD8+ T cells, B cells, Tregs, natural killer (NK) cells, NK T cells, conventional dendritic cells, plasmacytoid dendritic cells, and MDSCs) as well as 114 subsets relating to their maturation/function, as previously described [1,2].

*Circulating tumor cells*

Whole blood was collected in Streck (Omaha, NE) 10 mL Cell-Free DNA blood collection tubes. Samples were kept at room temperature and shipped on day of collection with ambient gel packs to Epic Sciences, Inc. (San Diego, CA) for CTC analyses, as previously described [3].

**Pharmacokinetics**

Modeling of the concentration-time profiles of HuMax-IL8 was performed using compartmental methods. All measured concentrations over time were utilized for each patient with determination of individual pharmacokinetic parameters. The following parameters were determined: elimination rate constant (K10), rate constant for transfer from central to peripheral compartment (K12), rate constant for transfer from peripheral to central compartment (K21), central volume of distribution (V1), peripheral volume of distribution (V2), volume of distribution at steady-state (Vss), systemic clearance (CL), first observed half-life after C_max_ (α-HL), and second observed half-life after C_max_ (β-HL). Pharmacokinetic calculations were performed using WinNonlin Professional Version 6.3 under Phoenix 64 (Certara L.P., Princeton, NJ). Analyses were based on actual times of dose administration and plasma sampling.

**References**

1. Donahue RN, Lepone LM, Grenga I, Jochems C, Fantini M, Madan RA, et al. Analyses of the peripheral immunome following multiple administrations of avelumab, a human IgG1 anti-PD-L1 monoclonal antibody. J Immunother Cancer. 2017;5(20.

2. Lepone LM, Donahue RN, Grenga I, Metenou S, Richards J, Heery CR, et al. Analyses of 123 peripheral human immune cell subsets: defining differences with age and between healthy donors and cancer patients not detected in analysis of standard immune cell types. J Circ Biomark. 2016;5:5.

3. Lu D, Graf RP, Harvey M, Madan RA, Heery C, Marte J, et al. Detection and characterization of circulating tumour cells from frozen peripheral blood mononuclear cells. J Circ Biomark. 2015;4:4.

**Table S1.** Patient demographics

| Baseline characteristic | DL 1  4 mg/kg  N = 3 | | DL 2  8 mg/kg  N = 3 | | DL 3  16 mg/kg  N = 3 | DL 4  32 mg/kg  N = 6 | Overall  N = 15 |
| --- | --- | --- | --- | --- | --- | --- | --- |
| Age (years)  Median (range) | 67 (59-71) | | 59 (51-65) | | 54 (46-66) | 60.5 (39-73) | 59 (39-73) |
| Race (N, %) |  | |  | |  |  |  |
| White/Caucasian | 2 (67%) | | 3 (100%) | | 3 (100%) | 4 (67%) | 12 (80%) |
| Black | 1 (33%) | | 0 | | 0 | 1 (17%) | 2 (13%) |
| Pacific Islander | 0 | | 0 | | 0 | 1 (17%) | 1 (7%) |
| Gender (N, %) |  | |  | |  |  |  |
| Male | 3 (100%) | | 2 (67%) | | 2 (67%) | 2 (33%) | 9 (60%) |
| ECOG PS (N, %) |  | |  | |  |  |  |
| 0 | 3 (100%) | | 1 (33%) | | 1 (33%) | 3 (50%) | 8 (53%) |
| 1 | 0 (0%) | | 2 (67%) | | 2 (67%) | 3 (50%) | 7 (47%) |
| Tumor type (N, %) |  |  | |  | |  |  |
| Chordoma | 1 (33%) | 1 (33%) | | 2 (67%) | | 1 (17%) | 5 (33%) |
| Colorectal | 1 (33%) | 1 (33%) | | 0 (0%) | | 2 (33%) | 4 (27%) |
| Prostate | 1 (33%) | 0 (0%) | | 0 (0%) | | 1 (17%) | 2 (13%) |
| Esophageal | 0 (0%) | 1 (33%) | | 0 (0%) | | 0 (0%) | 1 (7%) |
| Chondrosarcoma | 0 (0%) | 0 (0%) | | 1 (33%) | | 0 (0%) | 1 (7%) |
| Papillary thyroid | 0 (0%) | 0 (0%) | | 0 (0%) | | 1 (17%) | 1 (7%) |
| Ovarian | 0 (0%) | 0 (0%) | | 0 (0%) | | 1 (17%) | 1 (7%) |
|  |  |  | |  | |  |  |
| Number of prior therapies | Pt #1: 5  Pt #2: 1  Pt #3: 4 | Pt #4: 7  Pt #5: 3  Pt #6: 1 | | Pt #7: 3  Pt #8: 2  Pt #9: 3 | | Pt #10: 2  Pt #11: 2  Pt #12: 3  Pt #13: 5  Pt #14: 1  Pt #15: 1 | Median: 3  Range: 1-7 |

Baseline characteristics by dose level.

DL, dose level; ECOG PS, Eastern Cooperative Oncology Group Performance Score; kg, kilogram; mg, milligram; Pt, patient

**Table S2.** Pharmacokinetic analyses

**a**

| **Cycle 1, Day 1** | | | | | | | |
| --- | --- | --- | --- | --- | --- | --- | --- |
| **Parameter statistic** | **Treatment dose** | | | | | | |
|  | **4 mg/kg** | | **8 mg/kg** | | **16 mg/kg** | **32 mg/kg** | |
| Cmax (ug/mL)  Geo. Mean (N)  (% CV) | 89.3 (3)  (17) | 137 (3)  (17) | | 274 (3)  (3) | | 743 (6)  (12) | |
| Tmax (H)  Median (N)  (Min – Max) | 2.00 (3)  (1.23 – 4.88) | 2.00 (3)  (1.92 – 2.07) | | 2.00 (3)  (1.93 – 5.02) | | 2.08 (6)  (1.98 – 5.00) | |
| AUC (0 – T) (H*ug/mL)  Geo. Mean (N)  (% CV) | 5509 (3)  (97) | 13884 (3)  (66) | | 28192 (3)  (66) | | 67857 (6)  (54) | |
| AUC (tau) (H*ug/mL)  Geo. Mean (N)  (% CV) | N/A (0)  (N/A) | 23651 (2)  (19) | | 49926 (2)  (9) | | 110014 (4)  (10) | |
| Ctau (ug/mL)  Geo. Mean (N)  (% CV) | 28.4 (1)  (N/A) | 42.4 (2)  (0) | | 89.5 (2)  (6) | | 173 (4)  (15) | |
| **a**  **b**  **Cycle 1, Day 15** | | | | | | | |
| **Parameter statistic** | **Treatment dose** | | | | | | |
|  | **4 mg/kg** | | **8 mg/kg** | | **16 mg/kg** | **32 mg/kg** | |
| Cmax (ug/mL)  Geo. Mean (N)  (% CV) | 113 (3)  (12) | 201 (3)  (24) | | 434 (3)  (24) | | | 1013 (6)  (12) |
| Tmax (H)  Median (N)  (Min – Max) | 2.00 (3)  (1.08 – 2.00) | 2.22 (3)  (2.00 – 3.50) | | 2.02 (3)  (2.00 – 2.05) | | | 4.13 (6)  (2.05 – 5.00) |
| AUC (0 – T) (H*ug/mL)  Geo. Mean (N)  (% CV) | 19143 (3)  (9) | 31792 (3)  (12) | | 40934 (3)  (70) | | | 159934 (6)  (12) |
| AUC (tau) (H*ug/mL)  Geo. Mean (N)  (% CV) | 19143 (3)  (9) | 31792 (3)  (12) | | 78735 (2)  (8) | | | 159934 (6)  (12) |
| Ctau (ug/mL)  Geo. Mean (N)  (% CV) | 34.6 (3)  (20) | 61.2 (3)  (14) | | 144 (2)  (11) | | | 272 (6)  (25) |

Noncompartmental analysis was performed to characterize pharmacokinetic parameters for HuMax-IL8 on cycle 1, day 1 (**a**) and cycle 1, day 15 (**b**). Both the maximum concentration and area under the curve geometric mean of HuMax-IL8 demonstrated linear increases in exposure for the tested doses.

AUC, area under the curve; Geo, geometric; H, hours; Min, minimum; Max, maximum; Cmax, maximum concentration; Tmax, time to maximum concentration; Ctau, trough concentration

**Table S3. Immune subset analyses**


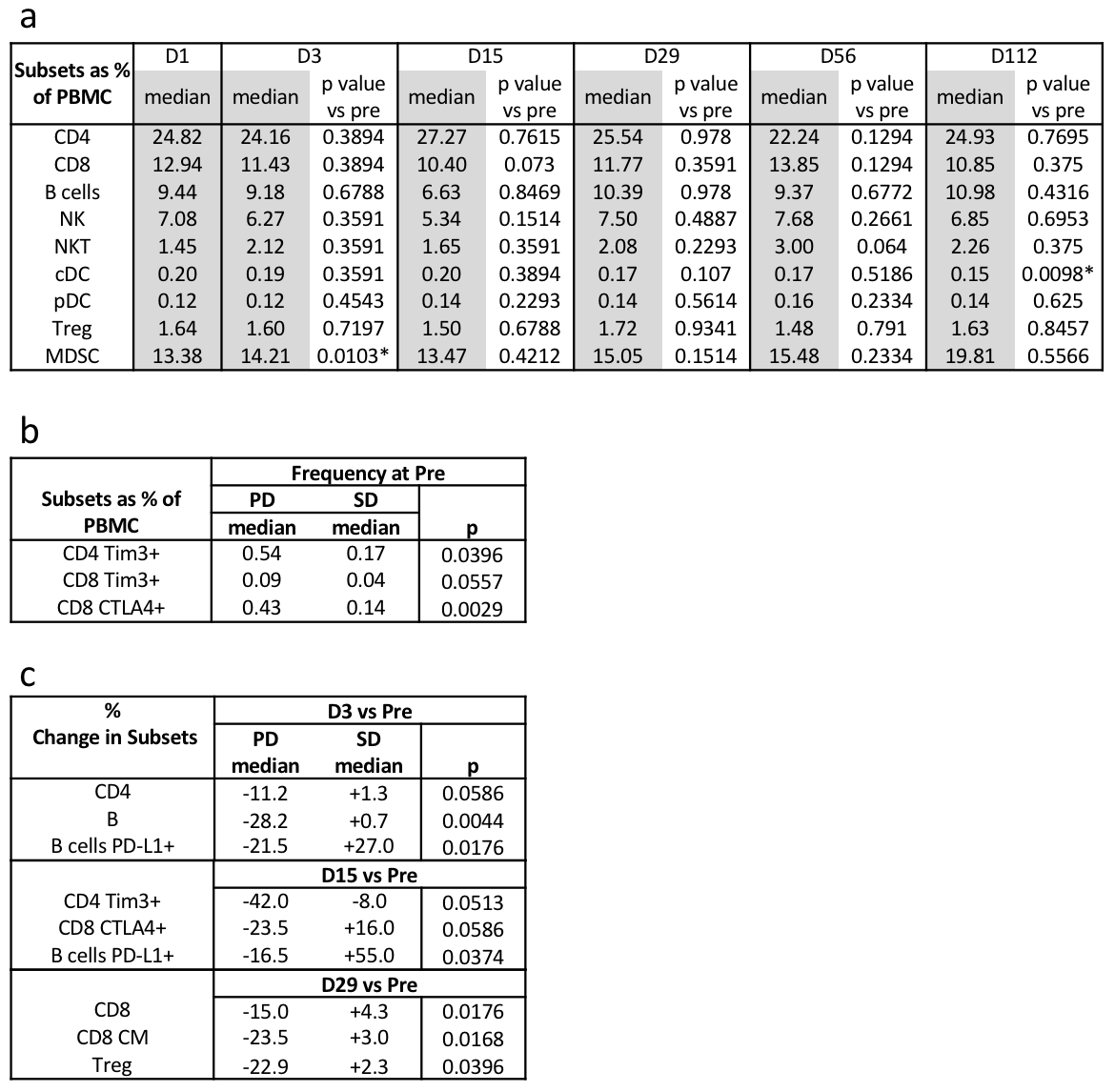


**a** HuMax-IL8 did not have a significant effect on PBMC subsets; data is shown for nine classic subsets. The p value was calculated using Wilcoxon matched pairs signed rank test. Criteria of significant change: *p* < 0.05, majority of patients > 25% change, differences in medians of pre- vs. post-therapy > 0.01% of PBMCs, and a frequency > 0.01% of PBMCs. *Although this p value is significant, the majority of patients did not change by > 25%. **b** The frequency of PBMC subsets at baseline are shown separated by best overall response of PD and SD; the subsets display a trend toward association with best overall response. The p value was calculated using the Mann-Whitney test. **c** Percent change in PBMC subsets is shown separated by best overall response of PD and SD; the subsets shown display a trend toward association with best overall response. The p value calculated by Mann-Whitney test.

CM, central memory cell; cDC, classical dendritic cell; CTLA-4; cytotoxic T lymphocyte-associated antigen 4; MDSC, myeloid derived suppressor cell; NK, natural killer; NKT, natural killer T cell; PBMC, peripheral blood mononuclear cell; pDC, plasmacytoid dendritic cell; PD-L1, programmed death-ligand 1; PD, progressive disease; Treg, regulatory T cell; SD, stable disease; Tim3, T cell immunoglobulin mucin 3

**Figure S1**

**
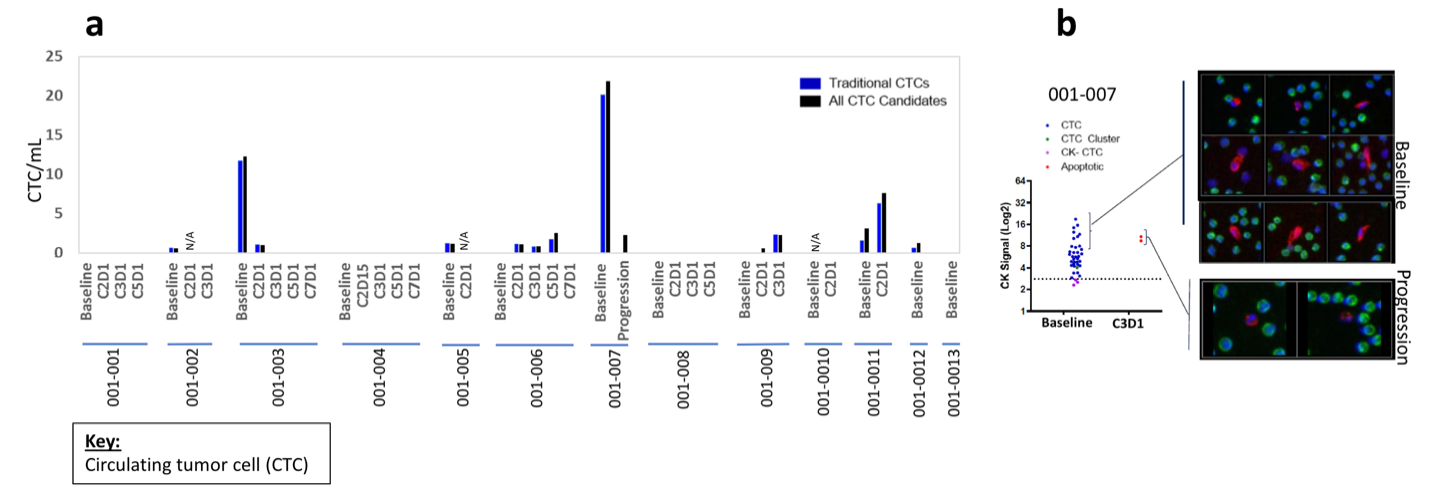
A** **B**

**Figure S1** Circulating tumor cell (CTC) enumeration. **a** Twelve patients had evaluable whole blood samples for CTC analysis. Two patients had CTCs > 10 CTC/mL at baseline, which then decreased significantly. **b** The immunofluorescence image shows that in patient #7, the CTCs remaining at progression were apoptotic.
